# Unique geometry of sister kinetochores in human oocytes during meiosis I may explain maternal age-associated increases in chromosomal abnormalities

**DOI:** 10.1242/bio.016394

**Published:** 2016-01-15

**Authors:** Jessica Patel, Seang Lin Tan, Geraldine M. Hartshorne, Andrew D. McAinsh

**Affiliations:** 1 Warwick Medical School, University of Warwick, Coventry CV4 7AL, UK; 2 Department of Obstetrics and Gynecology, McGill University, Montreal, Quebec H3A 1A1, Canada; 3 University Hospitals Coventry and Warwickshire NHS Trust, Coventry CV2 2DX, UK; 4 Centre for Mechanochemical Cell Biology, Warwick Medical School, University of Warwick, Coventry CV4 7AL, UK

**Keywords:** Aneuploidy, Chromosome segregation, Human, Kinetochore, Meiosis, Oocyte

## Abstract

The first meiotic division in human oocytes is highly error-prone and contributes to the uniquely high incidence of aneuploidy observed in human pregnancies. A successful meiosis I (MI) division entails separation of homologous chromosome pairs and co-segregation of sister chromatids. For this to happen, sister kinetochores must form attachments to spindle kinetochore-fibres emanating from the same pole. In mouse and budding yeast, sister kinetochores remain closely associated with each other during MI, enabling them to act as a single unified structure. However, whether this arrangement also applies in human meiosis I oocytes was unclear. In this study, we perform high-resolution imaging of over 1900 kinetochores in human oocytes, to examine the geometry and architecture of the human meiotic kinetochore. We reveal that sister kinetochores in MI are not physically fused, and instead individual kinetochores within a pair are capable of forming independent attachments to spindle k-fibres. Notably, with increasing female age, the separation between kinetochores increases, suggesting a degradation of centromeric cohesion and/or changes in kinetochore architecture. Our data suggest that the differential arrangement of sister kinetochores and dual k-fibre attachments may explain the high proportion of unstable attachments that form in MI and thus indicate why human oocytes are prone to aneuploidy, particularly with increasing maternal age.

## Abstract (precis)

**Summary:** Sister kinetochores in meiosis I human oocytes are not physically fused, with the degree of separation increasing with maternal age. This may have implications for the high incidence of aneuploidy in human oocytes.

## INTRODUCTION

The chances of a chromosomally abnormal pregnancy increase dramatically in humans with advancing maternal age (Nagaoka et al., 2012). Most meiosis-derived aneuploidies in early embryos originate from the first meiotic division of the oocyte, which is particularly error-prone (Hassold and Hunt, 2001). During the first meiotic division, sister chromatids segregate together, which requires kinetochores on sister chromatids to form attachments to spindle kinetochore-fibres (k-fibres) from the same pole of the spindle. This is in contrast to mitosis and meiosis II (MII), in which sisters form attachments to opposite spindle poles. In meiosis I (MI), therefore, it follows that the arrangement of sister kinetochores will be different; a side-by-side rather than the usual ‘back-to-back’ arrangement is likely (Watanabe, 2012). Of the meiotic sister kinetochores that have been studied so far, in maize, yeast and mouse, all appear to be in close association with each other, appearing as a single coherent unit. In maize and yeast, there is evidence that sisters are physically tethered: in maize, a Mis12-Ndc80 bridge links sisters (Li and Dawe, 2009); and in budding yeast, the monopolin complex performs a similar cross-linking role (Corbett et al., 2010; Sarangapani et al., 2014). In mouse oocytes, the meiotic regulator protein Meikin is important for keeping sister kinetochores together; loss of Meikin results in separation of sister kinetochores from a single unit into two distinct foci (Kim et al., 2015). A similar effect occurs in oocytes of aged mice, which is likely to reflect a loss of centromeric cohesin (Chiang et al., 2010). In human oocytes, the structure of the meiotic kinetochore is largely unknown with an initial study suggesting that inter-sister distances may increase in aged human oocytes (Sakakibara et al., 2015). It possible that an altered kinetochore geometry contributes to the features of human MI that differ from other species including the much higher incidence of aneuploidy and the protracted spindle assembly period (Holubcova et al., 2015).

## RESULTS

**Fig. 1.**
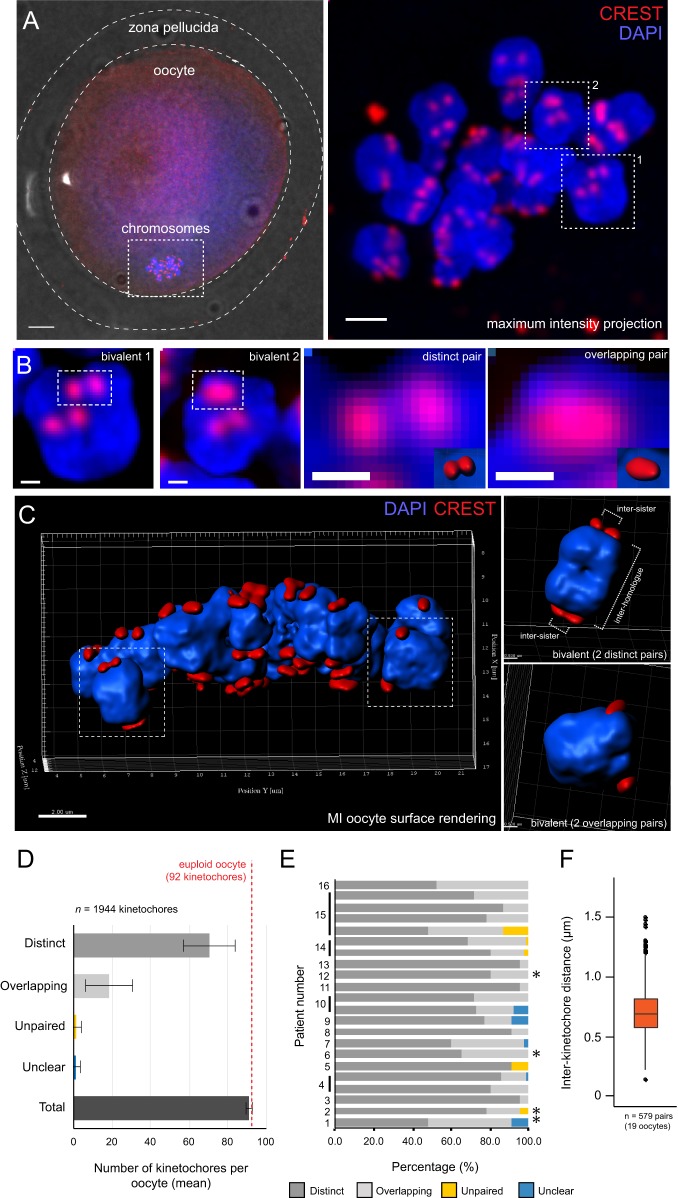
**Sister kinetochores in meiosis I (MI) human oocytes are not fused.** (A) Left panel: fixed human oocyte in MI. Dotted lines mark zona pellucida, oocyte and chromosomes (60× objective; scale bar=5 µm). Right panel: maximum intensity projection of the meiotic chromosomes within this same oocyte (100× objective; scale bar=2 µm). (B) Enlarged bivalents outlined in A, right panel, in which two kinetochore pairs per bivalent can be seen. Further enlargements of representative examples of distinct and overlapping sister kinetochore pairs are shown. Scale bars=0.5 µm. (C) 3D reconstruction of the kinetochores and chromosomes in an MI oocyte by surface rendering. To the right are two examples of individual bivalents showing the two categories of kinetochore pairs. (D) Mean±s.d. number of kinetochores within each oocyte, classified according to whether kinetochores were within distinct or overlapping pairs (*n*=1944 kinetochores from 22 oocytes). For a small number of kinetochores, sister kinetochores were so far apart (>1.5 µm) that they were classified as ‘unpaired’. A small number of foci could not be reliably identified as being either single kinetochores or overlapping pairs and these were classified as ‘unclear’. (E) Proportion of distinct and overlapping pairs for each individual oocyte. Asterisks indicate oocytes from women with no known fertility issues. (F) Inter-kinetochore distance as measured from the CREST signal (*n*=579 sister kinetochore pairs from 19 oocytes). Box plot represents interquartile range (IQR); whiskers extend to most extreme value within 1.5×IQR.

To investigate the geometry of sister kinetochores in MI, we examined sister kinetochore pairs in MI oocytes from women undergoing assisted reproduction following ovarian stimulation (Table S1). Our knowledge of mammalian meiosis is mostly based on mouse oocytes, because immature human oocytes available for research are typically only those that are not suitable for use in the donating patient's fertility treatment. However, it has been shown that the majority of clinically discarded immature human oocytes are able to undergo anaphase and exhibit consistent patterns of spindle assembly and chromosome segregation (Holubcova et al., 2015), highlighting their usefulness as tools for understanding human female meiosis. We therefore used human oocytes that had not yet completed the first meiotic division, confirmed by the absence of a polar body (Fig. 1A). Oocytes were fixed in paraformaldehyde and immunofluorescence was performed with CREST antisera to mark the centromere/inner kinetochore and DAPI to visualise chromosomes. High-resolution 3D image stacks (250×50 nm *z*-sections) of the meiotic chromosomes and kinetochores were collected using spinning-disk confocal microscopy. The number of CREST foci within these oocytes was considerably higher than 46, the number of kinetochores expected in a euploid MI oocyte in which all sister kinetochores are fused. This therefore raised the possibility that sister kinetochores are not fused. To investigate this, we marked sister kinetochore pairs in 3D image stacks, then classified these pairs on the basis of whether they appeared as ‘distinct’ pairs (two distinct spots) or ‘overlapping’ pairs (a single spot) (Fig. 1B). To confirm identity of pairs, we used surface rendering in three dimensions, which made it possible to identify individual bivalents associated with two sister kinetochore pairs (Fig. 1C). Classification was performed using *z*-projection images incorporating 20 *z*-sections (1.0 µm) centred about marked pairs (Fig. S1).

The majority (78%) of sister kinetochore pairs were identified as being distinct, with an average of 35 (range: 22–44) distinct pairs per oocyte (*n*=22 oocytes, Fig. 1D). A euploid oocyte has 46 kinetochore pairs in total, so distinct pairs account for the majority of kinetochore pairs in the population of oocytes we studied. The remaining 22% of sister kinetochore pairs were classified as overlapping, with an average of 10 (range: 0–22) overlapping pairs per oocyte. There were no differences in oocytes from women with no known fertility issues, i.e. couples with male factor infertility (marked with asterisks in Fig. 1E; see also Table S1), indicating that these observations are unlikely to result from infertility issues. To quantify the degree of separation, we used CREST signals to measure the inter-kinetochore distance between distinct sister pairs (see Fig. 1C, upper right panel). Distances were measured in 3D using the peak intensity of the kinetochore signal to mark individual kinetochore positions. The median inter-kinetochore distance was 0.69±0.21 µm (median±s.d.; *n*=579 pairs from 19 oocytes; Fig. 1F).

**Fig. 2.**
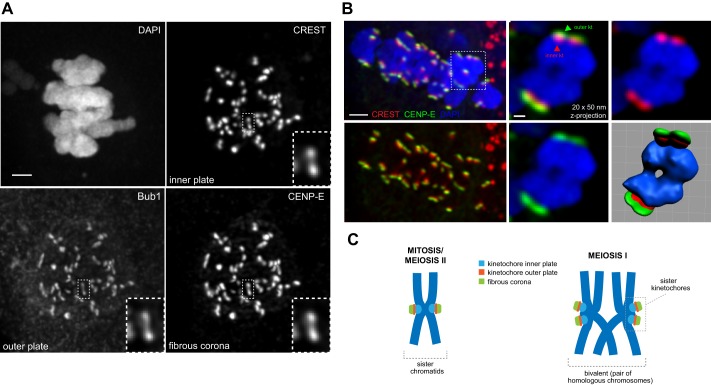
**Inner/outer/corona regions of each sister kinetochore are distinct.** (A) Chromosomes in a meiosis I (MI) oocyte stained with CREST antisera (kinetochore inner plate/centromere), anti-Bub1 antibodies (kinetochore outer plate) and anti-CENP-E antibodies (fibrous corona). Image is a maximum intensity projection incorporating 100×50 nm *z-*sections (5.0 µm). Inset shows a distinct sister kinetochore pair. Scale bar=2 µm. (B) Left upper and lower panels show a maximum intensity projection (100 × 50 nm *z*-sections) of chromosomes in an MI oocyte stained for CREST, CENP-E and DAPI. Lower-left panel shows the kinetochores only, in which CREST is clearly located towards the centromeric chromatin, with CENP-E on the outside. A projection (20 × 50 nm *z*-sections) of the outlined bivalent chromosome is shown in the middle and right panels, in which the arrangement of the inner (CREST, red) and outer (CENP-E, green) kinetochore can be seen more clearly. The bottom right panel depicts the surface rendered bivalent. Scale bars=2 µm (left panel), 0.5 µm (right panel). (C) Schematic showing the arrangement of sister kinetochores in mitosis (back-to-back) and the proposed arrangement in meiosis I (side-by-side).

The kinetochore is a large multi-subunit structure, consisting of an inner plate, outer plate and fibrous corona (Chan et al., 2005). A possible arrangement for human meiotic sister kinetochores may involve distinct inner plates (as we observed through CREST staining) but fused outer plates, an architecture observed in maize MI (Li and Dawe, 2009). As the outer plate is involved in formation of stable k-fibre attachments (Sundin et al., 2011), this model would enable sisters to form a single k-fibre attachment between them to ensure co-segregation. To test whether this model applies in humans, we used immunofluorescence to label the inner plate/centromere (CREST), outer plate (Bub1) and the fibrous corona (CENP-E) in oocytes (Wan et al., 2009; Earnshaw, 2015). Strikingly, the outer kinetochore markers also appeared separated in MI sister kinetochore pairs (Fig. 2A), demonstrating that the entire kinetochore structure is distinct. By fixing whole intact oocytes, it was also possible visualise the side-by-side arrangement of sister kinetochores, and show that the inner kinetochore (CREST) is located towards the centromeric chromatin, with the outer kinetochore (CENP-E) facing outwards (Fig. 2B,C). This indicates that the overall kinetochore architecture appears to be similar to that found in mitosis. Together, this data indicates that human MI sister kinetochores do not appear to be fused.

**Fig. 3.**
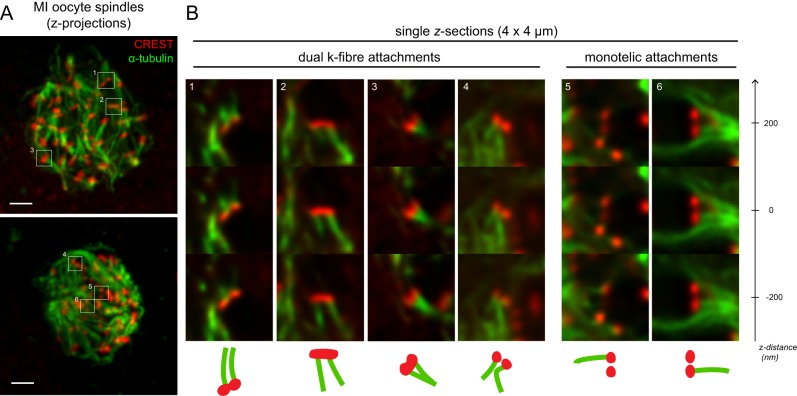
**Sister kinetochore pairs in meiosis I (MI) engage with independent kinetochore-fibres.** (A) Two MI human oocyte spindles stained for microtubules (anti-α-tubulin) and kinetochores (CREST antisera) after cold-shock treatment. (B) Enlarged *z*-sections of the six different kinetochore pairs outlined by white boxes in A, with dual and monotelic attachments as indicated. Three *z*-sections from the stack are shown for each pair, at –200 nm, 0 nm and +200 nm. Scale bar=2 µm.

One possibility is that in human MI, only one sister kinetochore is active and the other is shut off. To test this, we examined kinetochore-microtubule attachments in oocytes subjected to cold-shock treatment, which destabilises microtubules that are not attached to kinetochores in an end-on configuration. In a small number of cases (*n*=5 sister kinetochore pairs from three oocytes), we observed pairs in which one kinetochore was attached to a k-fibre but its sister was not. However, more frequently, we noted the presence of kinetochore pairs with dual k-fibre attachments (*n*=20), in which each sister within the pair was attached to a distinct k-fibre (Fig. 3). These attachments were observed in both distinct (18/20) and overlapping (2/20) kinetochore pairs, thereby providing evidence that homologous chromosomes can connect to the meiotic spindle via two independent attachment sites.

**Fig. 4.**
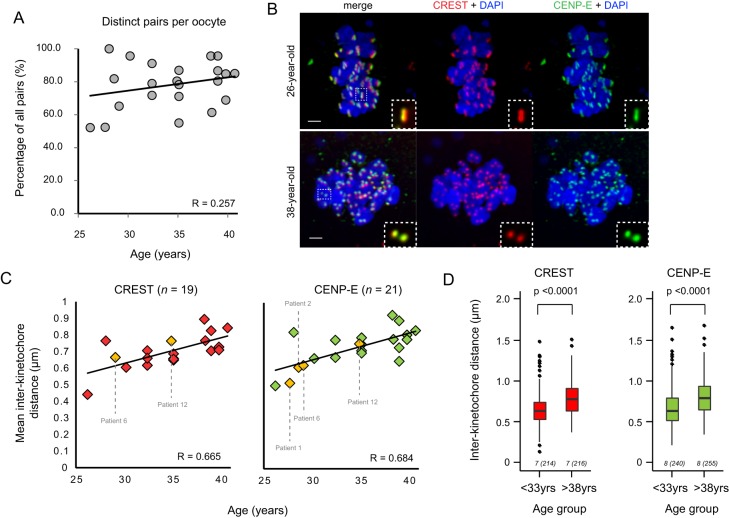
**Inter-kinetochore distance between sister kinetochores in meiosis I increases with maternal age.** (A) Relationship between proportion of distinct pairs per oocyte and female age (*n*=22 oocytes). (B) Comparison of oocyte chromosomes from a 26-year-old patient and a 38-year-old patient, showing increased inter-kinetochore distance with kinetochores marked with CREST antisera (red) and anti-CENP-E antibodies (green). Inset: representative example of a distinct kinetochore pair from each oocyte. Scale bars=2 µm. (C) Increasing inter-kinetochore distance with female age. Distance was measured in 3D from image stacks of kinetochore pairs, using CREST antisera (left plot) and anti-CENP-E antibodies (right plot) to mark the inner and outer regions of the kinetochores respectively. Patients with no known fertility problems (*n*=4) are marked in yellow. Patient numbers correspond to those shown in Table S1. R=linear correlation coefficient. (D) Comparison of inter-kinetochore distance between women under 33 years of age with women over 38 (*P*<0.0001, unpaired *t*-test) for CREST and CENP-E. Box plots represent interquartile range (IQR); whiskers extend to most extreme value within 1.5×IQR. The number of oocytes in each group is shown beneath each plot, with the total number of measurements in brackets.

During the first meiotic division, unlike in mitosis, cohesin is protected between sister kinetochores to ensure co-segregation (Kitajima et al., 2004). Cohesin loss over time has been the focus of many studies investigating maternal age-related aneuploidy (Jessberger, 2012). Therefore, we tested whether the high proportion of distinct pairs that we observed was associated with patient age. We found that there was no significant correlation between age and proportion of distinct pairs in our sample (Fig. 4A), although in the two youngest patients (26 and 27 years) just over 50% were separated, suggesting there may be a mild age-related effect. However, we noticed that in oocytes from older women, sister kinetochore pairs appeared to be further apart than in oocytes from younger patients (Fig. 4B). We therefore revisited our inter-kinetochore distance measurements and examined them in the context of age. Using both CREST and CENP-E as kinetochore markers, we found that there was a gradual increase in inter-kinetochore distance over the entire kinetochore structure with age (Fig. 4C). The subset of oocytes from women with no known fertility issues (*n*=4; labelled in Fig. 4C and see Table S1) also fit this trend suggesting this is part of the normal ageing process. As the majority of aneuploidies in human embryos arise when a woman is in her mid to late thirties (Hassold and Hunt, 2001), we compared oocytes from women under 33 years of age (age range: 26.2–32.4 years) with oocytes from women over 38 years of age (age range: 38.4–40.7 years). We found a significant increase in inter-kinetochore distance from a mean of 0.65±0.20 µm (*n*=214 pairs from seven oocytes) in women under 33 to 0.79±0.21 µm (*n*=216 pairs from seven oocytes) in those over 38 (*P*<0.0001, unpaired *t*-test) (Fig. 4D). We also compared these oocytes (under 33 years) with oocytes from women in their mid-thirties, for which the mean inter-kinetochore distance was 0.69±0.19 (*n*=149 pairs from five oocytes; age range: 34.9–35.1). We found that the difference in inter-kinetochore distance was not significant between the two younger groups of patients, but it was significant when comparing women in their mid-thirties with those over 38 years of age (*P*<0.0001). This is in keeping with the observed increase in MI-derived aneuploidy in oocytes from women in their mid to late thirties.

## DISCUSSION

In this study, we aimed to characterise the geometry of the meiotic kinetochore in human MI oocytes. Because distinct kinetochore pairs accounted for the majority of pairs in all oocytes from all women who donated to the study, this indicated to us that separated sister kinetochores are an intrinsic feature of human MI oocytes. This is different from yeast or mouse in which kinetochores are tightly held together during MI (Sarangapani et al., 2014; Kim et al., 2015), and plants in which only the outer plates of the kinetochores are fused (Li and Dawe, 2009). The human MI oocytes that we observed more closely resembled mouse Meikin-deficient oocytes, in which cohesin between sister kinetochores is no longer protected resulting in sister kinetochore separation (Kim et al., 2015). The variation in proportions of distinct and overlapping kinetochore pairs, even among oocytes from the same patient, may indicate a degree of compliance between sister kinetochores, as in mitosis (Jaqaman et al., 2010).

The ability of sister kinetochore pairs on a homologous chromosome to form dual k-fibre attachments indicates that both sisters are functionally active and are each capable of acting as independent attachment sites. We speculate that the presence of a monotelic population (one sister attached) may represent either an immature attachment or the result of an error-correction event. Clearly, the presence of four independent attachment sites could pose a significant problem for achieving stable co-orientation (both pairs of sister kinetochores attached to k-fibres from their respective spindle poles), particularly as the sister kinetochores move further apart. Furthermore, it means that there are potentially twice the number of kinetochore-microtubule attachments for the cell to correct. Given that data from both mouse and human oocytes indicate that the oocyte's ability to correct unstable kinetochore-microtubule attachments is less efficient than in mitosis (Yoshida et al., 2015), the larger number of possible connections may contribute to the increased spindle assembly time that is observed in human oocytes (Holubcova et al., 2015). This situation is dramatically different to what is known about mouse oocyte MI, in which the majority of sister kinetochores are held together by Meikin (Kim et al., 2015), thus forming a single k-fibre attachment (Kitajima et al., 2011; FitzHarris, 2012; Touati et al., 2015; Yoshida et al., 2015).

The absence of a correlation between age and the proportion of separated sister kinetochore pairs in human MI is different to what has been reported in mouse MI (Chiang et al., 2010). This is likely to reflect the fact that in our study population, the vast majority of kinetochores are separated, even in the youngest patients. No samples below 26 years of age were available because it is extremely rare for younger patients to have IVF. Nevertheless, the incidence of trisomic pregnancy is similar for women in their late teens/early twenties (2–3%) and their early thirties (∼5%), so we would not expect to see a dramatic difference in kinetochore geometry in women at earlier ages than those studied. In comparison, the incidence of trisomy for women in their late thirties/early forties is around 15% (Hassold and Hunt, 2001).

We show here that in intact human MI oocytes the entire kinetochore inner-to-outer-to-corona structure comes apart with age. This is in keeping with findings from monastrol-treated mouse MI oocytes (Chiang et al., 2010) and metaphase spreads from mouse and human MII oocytes (Merriman et al., 2013; Duncan et al., 2012). The maternal age-dependent change in centromeric chromatin may have implications for the formation of stable k-fibre attachments. One report in mouse has shown that separated sister kinetochores in aged oocytes do not form more unstable attachments than fused pairs, but they do have a slightly increased propensity to form merotelic attachments (Shomper et al., 2014), indicating that cohesin loss and subsequent separation of sisters does not severely affect attachment. However, given that the degree of separation of kinetochores in human oocytes is greater than that in mice, it may be a possibility that kinetochore pairs with an inter-kinetochore distance beyond a certain threshold are prone to mis-attachment, particularly as individual kinetochores within a pair act as separate attachment sites. It is also important to bear in mind that our results are only indicative of cohesin loss, and without direct study of cohesin there is the possibility that these changes in kinetochore geometry could be caused by other factors, such as changes in kinetochore or chromatin structure or microtubule-pulling forces.

In summary, our results provide a detailed insight into MI kinetochore geometry in intact human oocytes. We show that the majority of sister kinetochores in MI oocytes are separate, and that the degree of separation increases with age, consistent with the profile of maternal age-related aneuploidies in women. We also show that, as well as the chromatin-associated inner plate, the outer microtubule-interacting regions of the kinetochore are also separate, which facilitates the sister kinetochores acting as individual attachment sites. Since both sister kinetochores are able to form k-fibre attachments, stable bi-orientation may be more difficult to achieve, which may be exacerbated by increasing inter-kinetochore distances with increasing maternal age. These features of kinetochores in MI oocytes may shed light on the particularly high incidence of chromosome segregation errors at first meiosis in human oocytes.

## MATERIALS AND METHODS

### Donation of human oocytes to research

Approval for the project was granted by the NHS Research Ethics Committee (04/Q2802/26) and the Human Fertilisation and Embryology Authority (HFEA; Research Licence RO155). Informed consent for donation of oocytes to research was provided by patients undergoing *in vitro* fertilisation (IVF) or intracytoplasmic sperm injection (ICSI) at the Centre for Reproductive Medicine, University Hospitals Coventry and Warwickshire NHS Trust. All oocytes used for research were unsuitable for the patient's treatment and would otherwise have been discarded. For purposes of selection for research use, oocytes were presumed to be in MI if neither a germinal vesicle nucleus nor polar bodies were visible by light microscopy. This initial clinical assessment was further informed by detailed analysis of chromosomes in the course of the research.

### Whole oocyte fixation

Whole oocytes were fixed and stained using a method previously described (Riris et al., 2013). Briefly, oocytes were washed in PHEM buffer (60 mM PIPES, 25 mM HEPES, 10 mM EGTA, 4 mM MgSO_4_.7H_2_O; pH 6.9) with 0.25% Triton X-100, then fixed in 3.7% paraformaldehyde in PHEM for 30 min. Following fixation, they were washed in PBB (0.5% BSA in PBS), permeabilised with 0.25% Triton X-100 in PBS for 15 min, then transferred to a blocking solution (3% BSA in PBS with 0.05% Tween-20) where they were stored at 4°C overnight. For cold shock treatment, oocytes were placed in ice-cold media for 1 min immediately upon receipt, then fixation and immunofluorescence were performed as described.

### Immunofluorescence

Immunofluorescence was performed using a method previously described (Riris et al., 2013). Oocytes were incubated at 37°C for 1 h with primary antibodies diluted in blocking solution, followed by a 15 min wash in PBB with 0.05% Tween-20, then incubation with secondary antibodies for 1 h, followed by a final wash step. Primary antibodies included: anti-centromere antibody derived from human CREST serum (1:50; Antibodies Incorporated, Davis, CA, USA), rabbit anti-CENP-E (1:200; Meraldi et al., 2004) mouse monoclonal antibody against α-tubulin (1:200; T6074 Sigma-Aldrich, St Louis, MO, USA), mouse monoclonal antibody to Hec1 9G3 (1:50; ab3613 Abcam, Cambridge, UK) and mouse monoclonal antibody to Bub1 (1:50; Meraldi et al., 2004). Secondary tagged antibodies were diluted 1:200 and included anti-mouse Alexa Fluor 488^®^, anti-rabbit Alexa 594^®^ and anti-human Alexa 647^®^ (Stratech, Suffolk, UK). Oocytes were mounted in ProLong^®^ Gold Antifade Mountant with DAPI (Invitrogen, Carlsbad, CA, USA) for detection of chromosomes.

### Imaging

All imaging was performed on an UltraView spinning-disk confocal microscope (Perkin Elmer, Waltham, MA, USA). 3D image stacks were collected using a 60× NA1.4 (100×1 μm *z*-sections) and 100× NA1.4 (250×50 nm *z*-sections) oil objective. Images were acquired using Volocity software.

### Image and data analysis

Images were deconvolved using Huygens X11 (Scientific Volume Imaging B. V., Hilversum, Netherlands). Kinetochores were classified on the basis of their appearance in maximal projection images incorporating 10×50 nm *z*-sections above and below a manually marked point where the kinetochore/kinetochore pair appeared (covering a *z*-distance of 1.0 µm). To distinguish overlapping kinetochore pairs from single kinetochores, we used 3D reconstructions in Imaris (Bitplane, Zurich, Switzerland) to examine them in their chromosomal context. If foci could not be reliably classified, for instance due to overlapping chromosomes or kinetochore signal, they were classified as unclear. Inter-kinetochore distance was measured using the FindFoci plugin in ImageJ, which identifies regions of peak intensity in 3D image stacks (Herbert et al., 2014). For kinetochores in different *z*-sections, the Pythagorean formula was used to calculate inter-kinetochore distance. Statistical analysis of inter-kinetochore measurements was performed in Excel (Microsoft) or R (http://www.r-project.org/).

## References

[BIO016394C1] Chan G. K., Liu S.-T. and Yen T. J. (2005). Kinetochore structure and function. *Trends Cell Biol.* 15, 589-598. 10.1016/j.tcb.2005.09.010 16214339

[BIO016394C2] Chiang T., Duncan F. E., Schindler K., Schultz R. M. and Lampson M. A. (2010). Evidence that weakened centromere cohesion is a leading cause of age-related aneuploidy in oocytes. *Curr. Biol.* 20, 1522-1528. 10.1016/j.cub.2010.06.069 20817534 PMC2939204

[BIO016394C3] Corbett K. D., Yip C. K., Ee L.-S., Walz T., Amon A. and Harrison S. C. (2010). The monopolin complex crosslinks kinetochore components to regulate chromosome-microtubule attachments. *Cell* 142, 556-567. 10.1016/j.cell.2010.07.017 20723757 PMC2955198

[BIO016394C4] Duncan F. E., Hornick J. E., Lampson M. A., Schultz R. M., Shea L. D. and Woodruff T. K. (2012). Chromosome cohesion decreases in human eggs with advanced maternal age. *Aging Cell* 11, 1121-1124. 10.1111/j.1474-9726.2012.00866.x 22823533 PMC3491123

[BIO016394C5] Earnshaw W. C. (2015). Discovering centromere proteins: from cold white hands to the A, B, C of CENPs. *Nat. Rev. Mol. Cell Biol.* 16, 443-449. 10.1038/nrm4001 25991376

[BIO016394C6] Fitzharris G. (2012). Anaphase B precedes anaphase A in the mouse egg. *Curr. Biol.* 22, 437-444. 10.1016/j.cub.2012.01.041 22342753

[BIO016394C7] Hassold T. and Hunt P. (2001). To err (meiotically) is human: the genesis of human aneuploidy. *Nat. Rev. Genet.* 2, 280-291. 10.1038/35066065 11283700

[BIO016394C8] Herbert A. D., Carr A. M. and Hoffmann E. (2014). FindFoci: a focus detection algorithm with automated parameter training that closely matches human assignments, reduces human inconsistencies and increases speed of analysis. *PLoS ONE* 9, e114749 10.1371/journal.pone.0114749 25478967 PMC4257716

[BIO016394C9] Holubcova Z., Blayney M., Elder K. and Schuh M. (2015). Error-prone chromosome-mediated spindle assembly favors chromosome segregation defects in human oocytes. *Science* 348, 1143-1147. 10.1126/science.aaa9529 26045437 PMC4477045

[BIO016394C10] Jaqaman K., King E. M., Amaro A. C., Winter J. R., Dorn J. F., Elliott H. L., Mchedlishvili N., McClelland S. E., Porter I. M., Posch M. et al. (2010). Kinetochore alignment within the metaphase plate is regulated by centromere stiffness and microtubule depolymerases. *J. Cell Biol.* 188, 665-679. 10.1083/jcb.200909005 20212316 PMC2835940

[BIO016394C11] Jessberger R. (2012). Age-related aneuploidy through cohesion exhaustion. *EMBO Rep.* 13, 539-546. 10.1038/embor.2012.54 22565322 PMC3367239

[BIO016394C12] Kim J., Ishiguro K.-I., Nambu A., Akiyoshi B., Yokobayashi S., Kagami A., Ishiguro T., Pendas A. M., Takeda N., Sakakibara Y. et al. (2015). Meikin is a conserved regulator of meiosis-I-specific kinetochore function. *Nature* 517, 466-471. 10.1038/nature14097 25533956

[BIO016394C13] Kitajima T. S., Kawashima S. A. and Watanabe Y. (2004). The conserved kinetochore protein shugoshin protects centromeric cohesion during meiosis. *Nature* 427, 510-517. 10.1038/nature02312 14730319

[BIO016394C14] Kitajima T. S., Ohsugi M. and Ellenberg J. (2011). Complete kinetochore tracking reveals error-prone homologous chromosome biorientation in mammalian oocytes. *Cell* 146, 568-581. 10.1016/j.cell.2011.07.031 21854982

[BIO016394C15] Li X. and Dawe R. K. (2009). Fused sister kinetochores initiate the reductional division in meiosis I. *Nat. Cell Biol.* 11, 1103-1108. 10.1038/ncb1923 19684578

[BIO016394C16] Meraldi P., Draviam V. M. and Sorger P. K. (2004). Timing and checkpoints in the regulation of mitotic progression. *Dev. Cell* 7, 45-60. 10.1016/j.devcel.2004.06.006 15239953

[BIO016394C17] Merriman J. A., Lane S. I. R., Holt J. E., Jennings P. C., Garcia-Higuera I., Moreno S., McLaughlin E. A. and Jones K. T. (2013). Reduced chromosome cohesion measured by interkinetochore distance is associated with aneuploidy even in oocytes from young mice. *Biol. Reprod.* 88, 31 10.1095/biolreprod.112.104786 23255336

[BIO016394C18] Nagaoka S. I., Hassold T. J. and Hunt P. A. (2012). Human aneuploidy: mechanisms and new insights into an age-old problem. *Nat. Rev. Genet.* 13, 493-504. 10.1038/nrg3245 22705668 PMC3551553

[BIO016394C19] Riris S., Cawood S., Gui L., Serhal P. and Homer H. A. (2013). Immunofluorescence staining of spindles, chromosomes, and kinetochores in human oocytes. *Methods Mol. Biol.* 957, 179-187. 10.1007/978-1-62703-191-2_12 23138952

[BIO016394C20] Sakakibara Y., Hashimoto S., Nakaoka Y., Kouznetsova A., Hoog C. and Kitajima T. S. (2015). Bivalent separation into univalents precedes age-related meiosis I errors in oocytes. *Nat. Commun.* 6, 7550 10.1038/ncomms8550 26130582 PMC4507009

[BIO016394C21] Sarangapani K. K., Duro E., Deng Y., Alves F. d. L., Ye Q., Opoku K. N., Ceto S., Rappsilber J., Corbett K. D., Biggins S. et al. (2014). Sister kinetochores are mechanically fused during meiosis I in yeast. *Science* 346, 248-251. 10.1126/science.1256729 25213378 PMC4226495

[BIO016394C22] Shomper M., Lappa C. and Fitzharris G. (2014). Kinetochore microtubule establishment is defective in oocytes from aged mice. *Cell Cycle* 13, 1171-1179. 10.4161/cc.28046 24553117 PMC4013167

[BIO016394C23] Sundin L. J. R., Guimaraes G. J. and DeLuca J. G. (2011). The NDC80 complex proteins Nuf2 and Hec1 make distinct contributions to kinetochore-microtubule attachment in mitosis. *Mol. Biol. Cell* 22, 759-768. 10.1091/mbc.E10-08-0671 21270439 PMC3057701

[BIO016394C24] Touati S. A., Buffin E., Cladiere D., Hached K., Rachez C., Van Deursen J. M. and Wassmann K. (2015). Mouse oocytes depend on BubR1 for proper chromosome segregation but not for prophase I arrest. *Nat. Commun.* 6, 6946 10.1038/ncomms7946 25897860 PMC4439927

[BIO016394C25] Wan X., O'Quinn R. P., Pierce H. L., Joglekar A. P., Gall W. E., DeLuca J. G., Carroll C. W., Liu S.-T., Yen T. J., McEwen B. F. et al. (2009). Protein architecture of the human kinetochore microtubule attachment site. *Cell* 137, 672-684. 10.1016/j.cell.2009.03.035 19450515 PMC2699050

[BIO016394C26] Watanabe Y. (2012). Geometry and force behind kinetochore orientation: lessons from meiosis. *Nat. Rev. Mol. Cell Biol.* 13, 370-382. 10.1038/nrm3349 22588367

[BIO016394C27] Yoshida S., Kaido M. and Kitajima T. S. (2015). Inherent instability of correct kinetochore-microtubule attachments during meiosis I in oocytes. *Dev. Cell* 33, 589-602. 10.1016/j.devcel.2015.04.020 26028219

